# Music reduces pain and increases functional mobility in fibromyalgia

**DOI:** 10.3389/fpsyg.2014.00090

**Published:** 2014-02-11

**Authors:** Eduardo A. Garza-Villarreal, Andrew D. Wilson, Lene Vase, Elvira Brattico, Fernando A. Barrios, Troels S. Jensen, Juan I. Romero-Romo, Peter Vuust

**Affiliations:** ^1^Department of Neurology, Faculty of Medicine and University Hospital “Dr. Jose E. Gonzalez”, Universidad Autonoma de Nuevo LeonMonterrey, Mexico; ^2^Neuroscience Unit, Center for Research and Development in the Health Sciences (CIDICS), Universidad Autonoma de Nuevo LeonMonterrey, Mexico; ^3^Music in the Brain, Center of Functionally Integrative Neuroscience, Aarhus UniversityAarhus, Denmark; ^4^Faculty of Health and Social Sciences, School of Social, Psychological and Communication Sciences, Leeds Metropolitan UniversityLeeds, UK; ^5^Danish Pain Research Center, Aarhus University HospitalAarhus, Denmark; ^6^Department of Psychology and Behavioral Sciences, Aarhus UniversityAarhus, Denmark; ^7^Cognitive Brain Research Unit, Institute of Behavioral Sciences, University of HelsinkiHelsinki, Finland; ^8^Department of Music, Finnish Center of Excellence in Interdisciplinary Music Research, University of JyväskyläJyväskylä, Finland; ^9^Brain and Mind Laboratory, Department of Biomedical Engineering and Computational Science, Aalto University School of ScienceEspoo, Finland; ^10^Department of Behavioral and Cognitive Neurobiology, Institute of Neurobiology, Universidad Nacional Autonoma de Mexico Campus JuriquillaQueretaro, Mexico; ^11^General Hospital, Secretaria de Salud del Estado de QueretaroQueretaro, Mexico; ^12^Royal Academy of MusicAarhus, Denmark

**Keywords:** fibromyalgia, music, pain, analgesia, functional mobility

## Abstract

The pain in Fibromyalgia (FM) is difficult to treat and functional mobility seems to be an important comorbidity in these patients that could evolve into a disability. In this study we wanted to investigate the analgesic effects of music in FM pain. Twenty-two FM patients were passively exposed to (1) self-chosen, relaxing, pleasant music, and to (2) a control auditory condition (pink noise). They rated pain and performed the “timed-up & go task (TUG)” to measure functional mobility after each auditory condition. Listening to relaxing, pleasant, self-chosen music reduced pain and increased functional mobility significantly in our FM patients. The music-induced analgesia was significantly correlated with the TUG scores; thereby suggesting that the reduction in pain unpleasantness increased functional mobility. Notably, this mobility improvement was obtained with music played prior to the motor task (not during), therefore the effect cannot be explained merely by motor entrainment to a fast rhythm. Cognitive and emotional mechanisms seem to be central to music-induced analgesia. Our findings encourage the use of music as a treatment adjuvant to reduce chronic pain in FM and increase functional mobility thereby reducing the risk of disability.

## Introduction

Listening to music reduces acute and chronic pain (Guétin et al., 2012; Roy et al., 2012; Korhan et al., 2013). Several studies have suggested that the analgesic effect of music (or music-induced analgesia) may be secondary to cognitive and emotional effects that arise from listening to music: distraction from the pain, pleasantness, and pleasure, memory evoked emotions and relaxation (Mitchell et al., 2006; Juslin and Västfjäll, 2008; Roy et al., 2008, 2009; Wiech and Tracey, 2009; Bernatzky et al., 2011; Salimpoor et al., 2011). Distraction is a well-known cognitive analgesic mechanism (Tracey et al., 2002; Villemure and Bushnell, 2009) that is present when listening to music. Also, listening to music has been related to dopamine release from the caudate and the nucleus accumbens (Salimpoor et al., 2011), and dopamine itself is know to have a role in central analgesia (Wood, 2008). Pleasant (consonant) and unpleasant (dissonant) music has an effect on the descending pain modulation pathway, increasing or decreasing pain perception, respectively (Roy et al., 2009). Mitchell et al. (2006) found that familiarity with the music listened may be important to the analgesic effect and in a recent study we found that pleasant and relaxing nature sounds has the same analgesic effect as unfamiliar music (Garza Villarreal et al., 2012). Finally, the relaxation produced by music also influences the analgesic effect (Kenntnermabiala et al., 2007; Rhudy et al., 2008). Preferred or self-chosen music is likely to be especially proficient at reducing pain as self-chosen music is already liked, which provides an easily achievable sustained attention and entrainment (Mitchell and MacDonald, 2006; Mitchell et al., 2006). All this evidence suggests that music-induced analgesia may be regarded as a “central” type of analgesia, as the effect seems to occur in the brain stem, secondary to cognitive and emotional brain processes and by means of central neurotransmitters (i.e., dopamine), and not in the peripheral nociceptive receptors. Therefore, exploiting the features and elements of music that produce the analgesic effect should proficiently reduce pain in chronic pain diseases such as fibromyalgia, regarded as a generalized type of central pain.

Fibromyalgia (FM) is a chronic pain syndrome that is characterized by diffuse multifocal pain accompanied by fatigue, sleep disturbances, depression, cognitive, and memory issues, and alterations in sensory processing such as touch, audition, and vision (Wolfe et al., 1990; Brederson et al., 2011). FM patients also have significant limitations in functional mobility, namely difficulties in goal-directed movements due to generalized musculoskeletal pain (e.g., restrictions in tasks such as rising from a chair and walking), which in turn increase their risk of disability (Jones et al., 2010, 2012; Björnsdóttir et al., 2013). Functional mobility seems to improve with analgesia as shown in other chronic pain diseases such as low back pain (Taimela and Härkäpää, 1996; Evans et al., 2003). Nevertheless, in FM patients, functional mobility has only been measured in association to improvement due to physical exercise and training, not due to analgesia. Also, gait speed, part of functional mobility, is reduced with age and this should be taken into consideration (Alcock et al., 2013). Pain in FM is complicated and it is still not yet completely understood. These patients seem to suffer from a decreased central nociceptive input inhibition in the descending neural pathway that modulates pain (Petersel et al., 2010), meaning they seem to be more sensitive to pain and sensory inputs. Cognitively, FM patients catastrophize (thinking something is worse than it really is) more about pain than other chronic pain patients and show high levels of depression and anxiety (Hassett et al., 2000; Gracely et al., 2004; Thieme et al., 2004). FM may therefore at least in part be regarded as a central type of pain disorder and it can be hypothesized that FM patients would respond well to music-induced analgesia (Onieva-Zafra et al., 2010) and that their level of catastrophizing on pain may influence the efficacy of music on the FM patient.

The aims of the present study were: (1) to determine if slow-paced, self-chosen, highly pleasant music, regardless of the style reduces pain in FM; (2) to test if this music increases functional mobility in FM patients; (3) to determine if changes in functional mobility are related to pain and analgesia. We hypothesized that self-chosen music would reduce generalized pain in FM patients and consequentially increase their functional mobility.

## Materials and methods

### Patients

Twenty-two patients (21f/1m) diagnosed with FM, with an age between 22 and 70 years (median = 50) participated in the study. Prior to their participation the patients were interviewed about their clinical history, FM history, and medication. The patients were included in the study if they fulfilled the following criteria: (1) Diagnosis by a trained rheumatologist according to the American College of Rheumatology 1990 criteria for more than 1 year (Wolfe et al., 1990); (2) reported normal hearing and had no musical training; (3) were able to abstain from analgesic medication, structured exercise and alcohol consumption for at least 24 h, from caffeine for at least 12 h and from smoking for at least 2 h prior to the experiment. The exclusion criteria were: (1) Impossibility to walk; (2) morbid obesity; (3) uncontrolled endocrine diseases; (4) hearing deficiency; (5) pregnancy or lactation; (6) left-handedness; (7) MRI incompatibility (i.e., metal prosthetics). The patients were recruited at a FM help group from the city of Queretaro, Mexico. Table A1 shows the patients' comorbidities and medications. Written informed consent was obtained from all patients prior to the study, which was conducted in accordance with the Declaration of Helsinki. Patients received no compensation for taking part in the experiment. Ethical permission was obtained from Bioethics Committee of the Institute of Neurobiology, UNAM Juriquilla, Queretaro, Mexico.

### Experimental measures

Patients filled out the following questionnaires upon entrée into the study: the Spanish version of the Pain Catastrophizing Scale (PCS) (Sullivan et al., 1995; García Campayo et al., 2008) that measures thoughts and feelings when experiencing pain, State-Trait Anxiety Inventory (STAI) that assesses immediate (state) and general (trait) emotional, cognitive, and behavioral aspects of anxiety (Kendall et al., 1976), and the Center for Epidemiologic Studies Depression Questionnaire (CES-D) (Smarr and Keefer, 2011), a screening test for depression and depressive disorder. The STAI scores were used purely as descriptive variables in our population, whereas the PCS and CES-D scores were included in the analysis as variables that could affect pain perception, as well as each other.

Pain consist of a sensory and an emotional experience that can be measured subjectively using pain intensity (PI) and pain unpleasantness (PU) (Rainville et al., 1997; Coghill et al., 1999; Petzke et al., 2013). In our study, pain was measured using the Verbal Rating Scale (VRS) (Cork et al., 2004), in which the patients verbally reported their PI perception from 0 (no pain) to 10 (very intense pain) and PU from 0 (not unpleasant) to 10 (very unpleasant). Because we were interested in the effect of music-induced analgesia on functional mobility we also used the “Timed Up & Go” task (TUG) (Podsiadlo and Richardson, 1991), validated for quantifying general functional mobility in elderly population. This task focuses in basic mobility skills of the daily life necessary for ambulation such as, getting in and out of bed, on and off a toilet and walking a few feet. The TUG has been successfully used in FM patients in previous studies (Jones et al., 2002, 2010). To perform the TUG task, the patients were sitting comfortably in a chair, then they were asked to get up at the sound of the word “GO” and walk 3 m in a straight line, turn, walk back, and sit down again. The 3 m were marked on the floor by a tape. The time it took to complete the task was measured in seconds. Patients were not told the goal of the TUG to avoid bias as much as possible.

### Auditory stimuli and “wash-out”

Patients were presented with two auditory backgrounds: a control (pink noise) and a musical piece. The music piece was semi self-chosen music (see Procedure) that was highly pleasant and slower than 120 beats-per-minute (Table A2). The control and music piece lasted 10 min and were peak normalized using the open software Audacity (http://audacity.sourceforge.net/). Peak normalization is an automated process in which the software scans the entire signal to find the loudest peak, and then adjusts each sample to a specific level. It is used to ensure that the signal peaks at the loudest level allowed in a digital system and does not cause clipping in the sound system. To fit the music in 10 min, the patients had to listen to three songs, with the last one usually being cut short. The patients listened to the stimuli using Sennheiser HD 205 headphones with passive attenuation of ambient noise (© Sennheiser 2012 electronic GmbH & CO KG, Germany). To avoid any type of cognitive or analgesic “crossing” between auditory stimuli, a washout condition was used that consisted of watching and listening to a documentary chosen randomly by the experimenter from a pool of four different themes. We let the participant choose in order for them to actively attend to the video. The documentaries were about the history of the Mayans, the Aztecs, Egypt, and about Bill Gates. The washout condition lasted 10 min. The auditory stimuli and washout were presented using an Apple MacBook Pro laptop computer using the open-source software VLC Media Player (http://www.videolan.org).

### Procedure

The patients were recruited from a FM help group, where author JIRR gave a talk about new scientific findings in FM pain to the patients, specifically about cannabinoids. Author EAGV then announced about the scientific study of FM, without being specific as to the tasks involved and the main objective (music-induce analgesia). The volunteering patients where then added to a list for a following phone interview. In the phone interview the patients were screened for inclusion and exclusion criteria mentioned in the sub-section “Participants.” Then, we asked the patients for their favorite songs, musician, or band, taking into consideration that we wanted the songs to be pleasant and relaxing. The patients were asked for the names of at least three songs they wanted to listen to. We pre-screened the songs selected by the patients in order to ensure all songs in the experiment would have low beats-per-minute. The music chosen varied in cultural background and genre ranging from classical music to folk music; however, we chose music that had to ensure relaxation based on the patient's songs suggestions (Robb et al., 1995). The title and authors of the songs used in the study are listed in Table A2.

**Figure 1:**
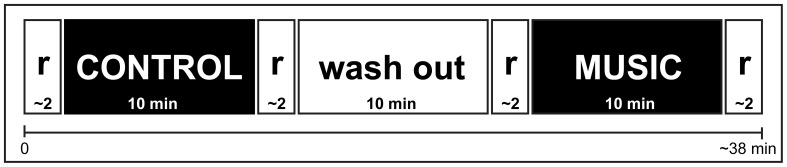
**Paradigm to study music-induced analgesia.** The participants listened to both conditions in between a washout period, with 2 min rests. r, Rest condition.

The study was performed in a well lighter soundproofed room with no windows, sat in a comfortable chair in front of a computer. The patients arrived to the lab and were briefed again about the study, they were told about their rights as participants, they filled out the psychological questionnaires and, if needed, were given extra time to relax. This allowed them to sit and rest for approximately 30 min before the experiment. Afterwards, the experiment began and it consisted of 10 min of one auditory background, 10 min of the washout condition, and 10 min of the other auditory background (Figure 1). After each auditory background listening, the patients rated their pain and functional mobility was tested. The presentation order of the auditory background was counterbalanced, meaning that half of the patients listened the control background first and the other half listened the music first. To avoid sleeping, closing their eyes or distraction, the patients were told to focus on the music and to fixate their eyes on a white cross with a black background that was presented in the computer. The experimenter (EAGV) was male and the same for all patients. During the study the experimenter was in another room adjacent to the patients' room, separated by a soundproof wall and a door.

### Statistical analysis

For the statistical analysis we used R Statistics (Team, 2011). We first performed descriptive statistics of the data. For the plots we used IBM SPSS version 20 and the ggplot2 package for R (Wickham, 2009). All variables were checked for normality using the Shapiro-Wilk's test (Shapiro and Wilk, 1965). Three variables were not normally distributed and the small sample size meant small power to reject normality. Therefore, we chose non-parametric two-tailed paired analyses using the Wilcoxon Signed Rank Test. We compared pre- vs. post-auditory stimulus for each condition (control and music) for each dependent variable (PI, PU, TUG). To determine if there was a positive relation between the analgesic effects and functional mobility, we performed one-tailed non-parametric correlations between the difference scores (post-, pre-auditory stimulus) of the PI, PU, and TUG and we represent these as ΔPI, ΔPU, and ΔTUG. Afterwards, we performed simple and multiple linear regression analyses to understand the relationship between the TUG (dependent), PI, PU, and “age” of the patients as independent variables. Age was introduced as a covariate because functional mobility should be affected by age of the participant as mentioned earlier. We also investigated correlations between the PCS and the PI, PU, and TUG variables. Finally, a two-tailed non-parametric correlation analysis between the order of the sampling and PI, PU, and TUG scores was performed to study a possible bias by an order effect (even after the counter-balancing). The alpha level for all statistical analyses was 0.05. Data were corrected for multiple comparisons using the false discovery rate (FDR) correction (Benjamini and Hochberg, 1995) and only corrected *p*-values are shown. Assumptions of non-zero variance, no multicollinearity, homoscedasticity, independence of errors, normality of errors, independence, and linearity were checked in all regression models.

## Results

**Figure 2:**
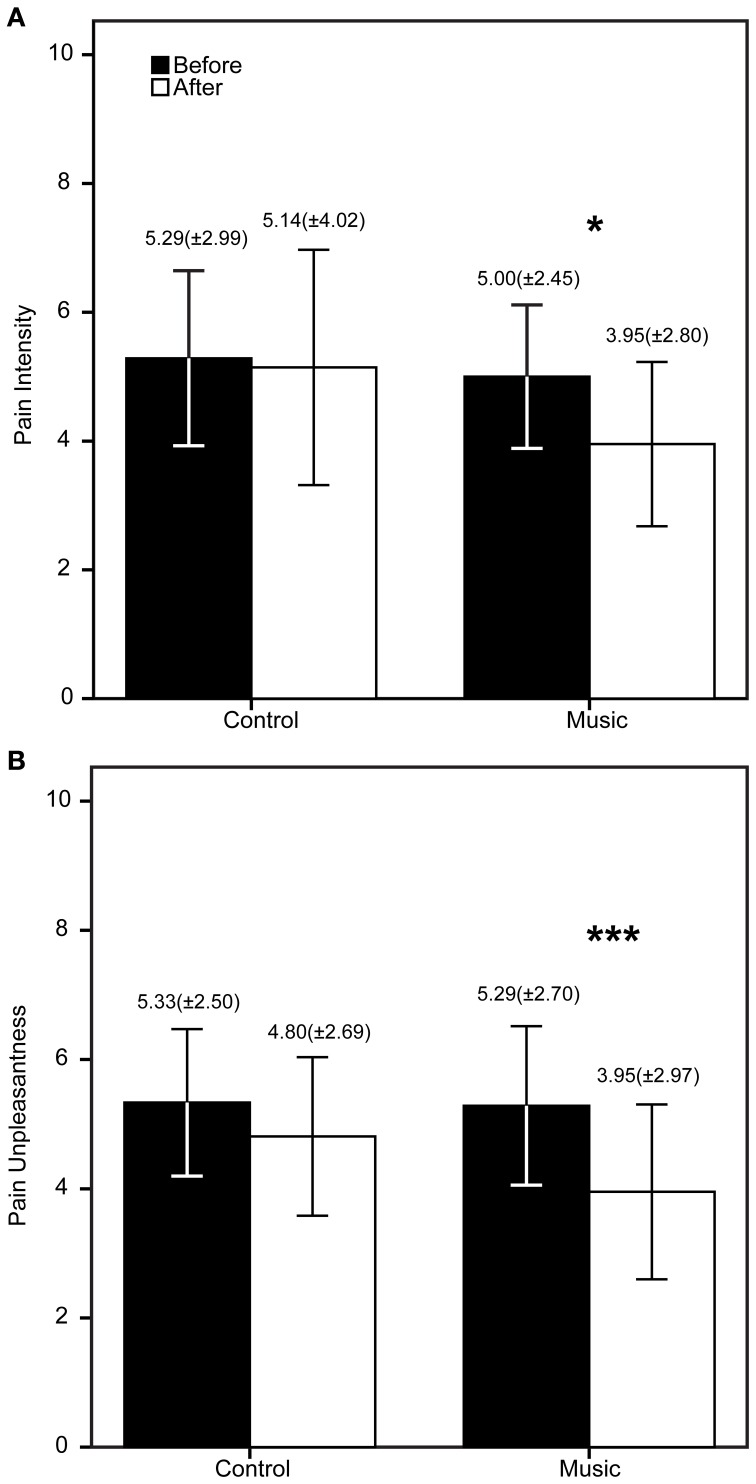
**Results of the verbal rating scale (VRS) before and after the control/music conditions.** The bars represent the mean of each measure (**A** = Pain intensity, **B** = Pain unpleasantness), whereas the error bars represent the standard deviation. Above the error bars we show the Mean (*SD*) for each measure. ^*^p < 0.05, ^***^p < 0.006.

**Figure 3:**
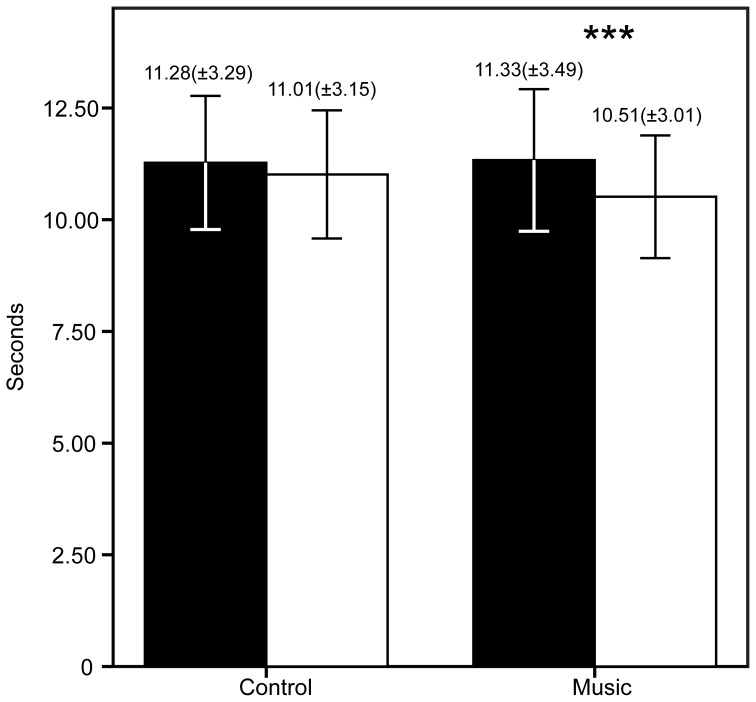
**Results of the timed up and go task (TUG) before and after the control/music conditions.** The bars represent the mean of each measure, whereas the error bars represent the standard deviation. Above the error bars we show the Mean (*SD*) for each measure. ^***^p < 0.006.

The patients demographic and questionnaire results are shown in Table 1. The FM patients show high scores in all questionnaires as expected in this population. The PCS showed high scores of catastrophizing, whereas the STAI showed medium to high scores of anxiety. The CES-D showed an average score of 25.43 (maximum = 49, minimum = 7), higher than 16, meaning that patients had signs of depression. To be precise, 80% of the FM patients had scores >16. The descriptive statistics of the PI, PU, and TUG are shown in Figures 2, 3. The patients reported significantly less pain only in the Music condition: PI (*Z* = −2.33, *p* = 0.04) and PU (*Z* = −2.85, *p* = 0.006). This means the patients reported feeling less pain after the music rather than after the control auditory stimulus. Furthermore, the patients were significantly faster after the Music condition in the TUG task (*Z* = −2.88, *p* = 0.006), but not after the Control condition.

**Table 1:** **Demographic information**.

**Variable**	**Mean**	***SD***
Age (median, range)	50	(22–70)
Pain catastrophizing scale	26.33	±13.13
State-trait anxiety inventory	51.20	±19.93
Center for epidemiology studies depression	25.43	±12.25

SD, standard deviation.

**Figure 4:**
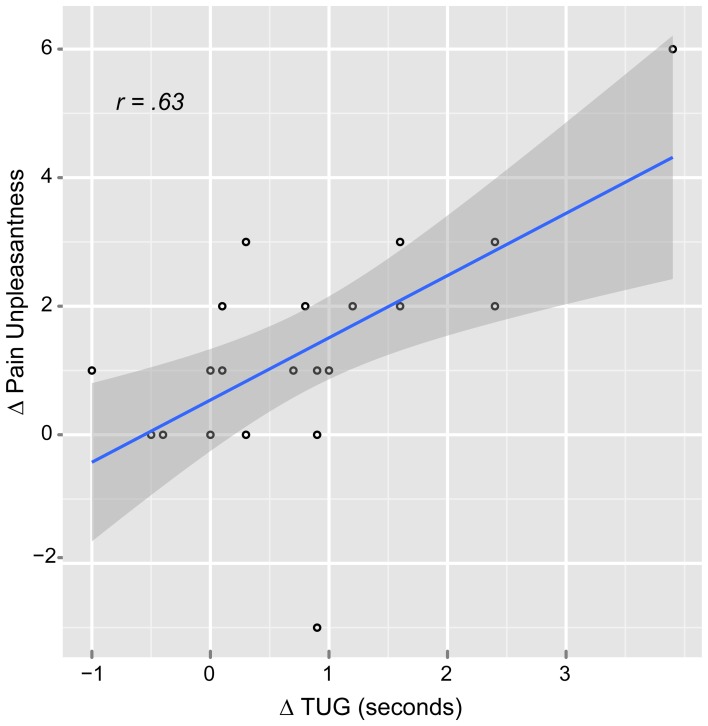
**Scatter plot of the relationship between pain unpleasantness and functional mobility difference scores in the music condition.** Δ, pre-post auditory stimulus; TUG, Timed up and go task.

The correlation analysis showed significant positive correlation only between the music ΔPU and ΔTUG (*r* = 0.63, *p* = 0.007) suggesting that when the patients reported less PU, they were also faster at the TUG task for functional mobility (Figure 4). There were also significant positive correlations between age and music: ΔPI (*r* = 0.61, *p* = 0.007) and ΔPU (*r* = 0.61, *p* = 0.009). This means that the older the patient the higher analgesic effect reported. Interestingly, there was no correlation between “age” and control ΔPI nor ΔPU.

The simple regression analysis (Table 2) showed an important relationship between music ΔTUG and both ΔPU and ΔPI, though the effect size in the music ΔTUG/ΔPU was greater. Therefore, the multiple regression analysis (also Table 2) was run between music ΔTUG, ΔPU, and age. ΔPI was left out because of probable multicollinearity with ΔPU and the smaller effect size. This model was the best to explain the variance of music ΔTUG, suggesting a strong relationship between pain and functional mobility when controlling for age. The order analysis showed no significant correlation between scores, which suggested that the scores were not affected by the order of the VRS and TUG. Finally, as Table 3 illustrates, the PCS scores were correlated to the control and music PI and TUG, however only for the music PU probably due to the multiple comparisons correction. The effect sizes were greater in the music condition than the control condition in general.

**Table 2:** **Simple and multiple linear regression analyses of the TUG**.

	***B***	***SE B***	**β**	***R*^2^**
**SIMPLE REGRESSION**				
Intercept	0.54	0.25		
PI (b1)	0.27	0.12	0.47^*^	
				0.22
Intercept	0.28	0.25		
PU (b1)	0.40	0.12	0.63^**^	
				0.39
**MULTIPLE REGRESSION**				
Intercept	1.91	0.86		
PU (b1)	0.57	0.14	0.89^***^	
Age (b2)	−0.04	0.02	0.43	
				0.51

B, b-values; SE B, Standard Error of B; Step 1, Single linear regression models; Step 2, Multiple linear regression model;

* p < 0.05,

** p < 0.01,

*** p < 0.001.

**Table 3:** **Correlations with Pain Catastrophizing Scale**.

**PCS**	***r***	***p*-value**
PIcb	0.46	0.017^*^
PIca	0.48	0.013^*^
PUcb	0.31	NS
PUca	0.30	NS
PImb	0.79	<0.001^*^
PIma	0.53	0.006^*^
PUmb	0.63	0.001^*^
PUma	0.48	0.014^*^
TUGcb	0.45	0.02^*^
TUGca	0.47	0.015^*^
TUGmb	0.44	0.02^*^
TUGma	0.54	0.005^*^

One-tailed Spearmann correlations (alpha 0.05), r, correlation coefficient. PCS, Pain Catastrophizing Scale; PI, pain intensity; PU, pain unpleasantness; TUG, Timed Up and Go task; c, control; m, music; b, before stimulus; a, after stimulus. Multiple comparisons corrected using FDR.

* , Variables that passed multiple comparisons.

## Discussion

In our study, music reduced pain and increased functional mobility significantly, and the functional mobility improvement seemed to be highly related to the music-induced analgesia. Appreciation of music depends heavily on cultural background and there is a great variety of musical styles. In terms of music-induced analgesia, we found that self-selected music is proficient to reduce pain. Our study supports previous findings of music-induced analgesia obtained with familiar music having a personal self-referential value. Mitchell et al. (2006) found that preferred music helped participants to tolerate pain longer than with music chosen by the experimenter. It is not yet known which are the specific mechanisms behind music-induced analgesia, however the evidence suggests that the intrinsic and extrinsic properties of the music should have an indirect influence on the effect via cognitive and emotional mechanisms (Roy et al., 2008, 2012; Bernatzky et al., 2011; Hauck et al., 2013). It is culturally and scientifically known that music is a powerful inducer of emotions and a modulator of mood (Baumgartner et al., 2006; Juslin and Västfjäll, 2008; Fritz et al., 2009; Bernatzky et al., 2011), and it is also known that emotions and mood can reduce pain (de Tommaso et al., 2008; Villemure and Bushnell, 2009). Music-induced analgesia then possibly acts by means of central mechanisms such as the release of neurotransmitters like dopamine, the regulation of the autonomic system (Salimpoor et al., 2011) and involvement of the anterior cingulate cortex (ACC) as an integrative hub between affect, pain, and cognition (Shackman et al., 2011). PI is regarded as a measure of nociception, the physical sensation of pain, whereas PU is regarded as a measure of the affective component of said pain (Rainville et al., 1997). Pain catastrophizing and affect seem to be an important part of the FM pain, and perhaps this is the reason for the greater effects in the affective dimension of pain than in the sensory dimension (Gracely et al., 2004). Our sample of FM patients showed high scores of pain catastrophizing that was correlated to the pain rating and also showed the highest analgesic effect on the PU dimension, suggesting a strong emotional component for the analgesic effect of music in this population.

From various studies there is evidence of several cognitive analgesic mechanisms: distraction, reward (Kringelbach, 2005), reappraisal and expectation of pain relief (Wiech et al., 2008). Active or passive distraction reduces pain perception possibly by affecting the periacqueductal gray (PAG) (Tracey et al., 2002), ACC, and posterior parietal cortex (PPC) (Villemure and Bushnell, 2009). Reward mechanisms in analgesia are related to dopamine and opioid release. Reappraisal and expectation of pain relief have been shown in relation to opioid receptors (Bingel and Tracey, 2008). Attributes such as familiarity with the music, pleasantness, and arousal, important elements for central analgesia, are strongly present in music and possibly activating the mesocorticolimbic dopamine pathway involved in reward and pleasure (Pereira et al., 2011; Salimpoor et al., 2013). All this evidence suggests that music-induced analgesia could be regarded as a “central” type of analgesia, derived from top-down mechanisms (Tracey, 2007). In our study we found that relaxing and highly pleasant self-chosen music reduced pain in FM patients. Although this was not directly measured, passive distraction, reward, reappraisal, and expectation of pain relief may be mechanisms present in the analgesic effect, perhaps due to the familiarity, pleasantness and sense of control that the music provided.

The music styles listened by the participants in this study varied from pop to folk to classical. There is a cultural belief that classical music is better to reduce pain the any other type, which was probably started or exacerbated by the so-called “Mozart effect” that is not even related to pain perception (Steele et al., 1999; McKelvie and Low, 2010). In a previous study we found that pleasant and relaxing nature sounds have the same analgesic effect as music when the music is not familiar, suggesting that the emotional and cognitive processing of music may be more important for the analgesic effect than the music *per se* (Garza Villarreal et al., 2012). Although in this FM study we did not test for the difference between classical and other music styles, we would like to point out that we show an analgesic effect with several types of music. Exploiting the analgesic mechanisms involved in the intrinsic properties of the music should produce analgesic effects in FM, as shown in our results, and reduce the individual variability present in pain studies that study music as an adjuvant for pain. Individual variability is an important issue in both music and FM studies. A recent review on music-based interventions in palliative cancer care shows a good example of the high variability between studies researching the use of music for chronic pain, anxiety, and mood (Archie et al., 2013). A controlled study in depression patients showed that individualized music therapy reduced depression, anxiety, and increased functioning significantly (Erkkila et al., 2011). FM is a complex disorder with different levels of reported pain in each individual. The reason for the variation of the pain is unknown, although it seems the modulation of the proposed central pain amplification may be mediated by NMDA receptors and nerve growth factor (NGF) (Petersel et al., 2010), as well as cognitive and emotional comorbidities such as depression (Smith et al., 2011).

Loss of mobility is a serious issue in FM, as it may lead to functional disability (Jones et al., 2010). Difficulty to perform complex activities such as working or self-care can be a great burden to the patient with FM and the family. Here we found increments in functional mobility after 10 min of listening to music, meaning the FM patients were faster to get up from a chair and walk. Motor entrainment and increases in gait speed secondary to rhythmic auditory cues have been shown in Parkinson's disease and other diseases (Lim et al., 2005; Wittwer et al., 2013). Even simple rhythmic beats like those from a metronome can provide cues for the brain to scale movement time, whereas complex sounds such as music can increase arousal, motivation, and rhythm perception (Karageorghis et al., 2009). However, our results were obtained with slow music (low beats-per-minute) before the motor task (not during), therefore the functional mobility effect cannot be explained merely by motor entrainment to a fast rhythm that may speed up walking. We also found that the increase in functional mobility was significantly and strongly correlated with the analgesia in music; this provides supporting evidence that the reported pain reduction may have been accurately and physically perceived. Despite the fact that the “timed up & go” task was initially developed to study functional mobility in elderly population (Podsiadlo and Richardson, 1991), it is proving to be a valuable tool for studying functional mobility in FM (Jones et al., 2002, 2012). In our results, the correlation between analgesia and functional mobility was significant only in the PU dimension. It could then be argued that music-induced analgesia mainly affects the emotional component of pain in FM and this is enough to overcome reduced functional mobility secondary to the chronic pain. A study found that FM patients with depressive symptoms report more pain and deficit in pain inhibition than those without depressive symptoms (de Souza et al., 2009). We found that our FM patients had high scores for depressive symptoms shown the CES-D and high catastrophizing shown by the PCS. Therefore, there seems to be a prominent affective component in FM that is related to the pain itself where music-induce analgesia may act and thus also improve functional mobility. Our current results suggest that to use music-induce analgesia in chronic pain patients it is necessary to take into consideration individual musical preference as well as other features such as pleasantness and beats per minute. However, the precise “amount of music” needed to induce an analgesic effect, the time it takes for the music to produce an analgesic effect, the duration of the analgesic effect, the internal and/or external agents that influence these properties are still unknown. As with any other medical treatment, it is important to answer these questions to deliver proper analgesic treatment.

One limitation of the study is that the TUG task is a general test and does not provide detailed information about motor function. Although motor function and gait could be studied using specialized methods such as motion capture, in our study the TUG was meant as a general measure of functional mobility in relation to analgesia. Another limitation is that FM is highly heterogeneous; (1) in the amount of pain the patients report, (2) in the co-morbidities such as depressive symptoms and (3) in the medication used. This could introduce noise in the data that could inflate the type II error (false negatives). Nevertheless, in our study the analgesic effect of music seems to be robust enough to overcome this. Most of the music in our study included lyrics, so it is not possible to separate the effect of the lyrics from the musical score. However, this was not the purpose of our study (although, see Brattico et al., 2011). We wanted to potentiate the analgesic effect by using music that people listen to in their daily life to ensure the music was highly pleasant and relaxing, and to induce an analgesic effect by all possible factors such as; familiarity, sense of control, among others. In this study, we regard music as the musical score and the lyrics. Another limitation is that the use of the not self-selected pink noise as the control condition does not allow separating the effect of controllability present in the self-selected music. Though, from other studies we know that controllability performs an important role in analgesia not only in music (Mitchell and MacDonald, 2006; Mitchell et al., 2006; Wiech et al., 2008) and it was not the purpose of the study to investigate it. Finally, we previously discussed that music can reduce depression and this effect could play a role in music-induced analgesia. In our study we did not investigate the effects of music on depression scale, however it would be interesting to study the effects of music on depression in relation to analgesia.

In this study we found that listening to music that is relaxing, highly pleasant, familiar, and self-chosen, reduced pain and increase functional mobility in fibromyalgia patients. We also found that the improvement in functional mobility was closely related to music-induced analgesia, especially in the affective dimension of the pain. Therefore, we suggest that music reduces pain in fibromyalgia by means of cognitive and emotional mechanisms and that this analgesic effect is strong enough to increase their functional mobility. We suggest that it is important to exploit the features of the music known to produce the strongest analgesic effect when it is used as an adjuvant for pain therapy.

### Conflict of interest statement

The authors declare that the research was conducted in the absence of any commercial or financial relationships that could be construed as a potential conflict of interest.

## Appendix

**Table A1:** **Medical history of the FM patients**.

**ID**	**Comorbidity**	**Medications**
1	Glaucoma	NSAID
	HPV infection	
2	Migraine	Pregabalin
	Allergic rhinitis	Procaine
		SSRI
	Vertigo	
3	Neurocardiogenic syncope	
	Cleft palate (treated)	
4	NR	TCA
		NSAID
5	NR	TCA
		NSAID
6	NR	Benzodiazepine
		Opioid analgesic
		TCA
		NSAID
7	Hypothyroidism	Fludcortisone
		SSRI
		Levothyroxine
		Melatonin
		Benzodiazepine
		Glucosamine-Chondroitin
8	NR	Gabapentin
		Valproate
		NSAID
		Ranitidine
		SSRI
9	NR	NR
10	NR	Benzodiazepine
11	Hypothyroidism	Levothyroxine
		Benzodiazepine
12	NR	Gabapentin
		NSAID
13	NR	Chondritin
		Eletriptan HBr
		Nabilone
15	NR	Opioid analgesic
		Aspirin (preventive)
		NSAID
		DMARD
17	HTN	TCA
		NSAID
18	HTN	TCA
	DM2	Losartan
	Hypothyroidism	Eletriptan HBr
	IBS	NSAID
	Migraine	Levothyroxine
19	HTN	Enalapril maleate
	Hypothyroidism	Levothyroxine
		TCA
		Cyclobenzaprine
		NSAID
20	NR	TCA
		Benzodiazepine
21	NR	Hormone replacement
		Glucosamine-Chondroitin
23	NR	NR

NR, Not reported; NSAID, Non-steroidal anti-inflammatory drug; SSRI, Selective serotonin reuptake inhibitor; TCA, Tricyclic antidepressant; DMARD, Disease-modifying antirheumatic drugs; HTN, Hypertension.

**Table A2:** **Music chosen by the participants**.

**Artist**	**Songs**
Barry Manilow	Looks like we made it—Mandy—The long and winding road
Joan Sebastian	Tatuajes—Secreto de Amor
Moby	Porcelain—If things were perfect—Everloving
Pearl Jam	I am mine—Elderly woman behind a counter in a small town—Nothingman
Luis Miguel	Somos novios—La media vuelta—La incondicional
Vicente Fernandez	La diferencia
Vivaldi	Four Seasons—Spring allegro pastorale largo—Autum allegro
La oreja de Van Gogh	Cuentame al oido
Enrique Bunbury	Frente a frente
Los Tigres del Norte	Golpes en el corazon
Miguel Bose	Si tu no vuelves—Mia
Wolfgang Amadeus Mozart	String Quartet No. in G major Adagio—Violin Concerto No. 3 in G major Adagio
Barry White	Come on—The right night
Pepe Aguilar	Me estoy acostumbrando a ti—Perdoname
Jose Jose	La nave del olvido—Amar y querer—La gloria eres tu
La quinta estacion	Sueños rotos
Michael Bolton	What won't you do for love—Whiter shade of pale—Ain't no sunshine when she's gone
Mike Rowland and Chris Mitchell	Dreamtime Dolphin—New Age
Yanni	The very best selection
Mike Rowland	This time
Cat Stevens	Morning has broken—How can I tell you—Into white
